# HS3ST3A1 and CAPN8 Serve as Immune-Related Biomarkers for Predicting the Prognosis in Thyroid Cancer

**DOI:** 10.1155/2022/6724295

**Published:** 2022-12-22

**Authors:** Zhao-Hui Chen, Hao-Ran Yue, Jun-Hui Li, Ruo-Yu Jiang, Xiao-Ning Wang, Xue-Jie Zhou, Yue Yu, Xu-Chen Cao

**Affiliations:** ^1^The First Department of Breast Cancer, Tianjin Medical University Cancer Institute and Hospital, National Clinical Research Center for Cancer, Tianjin 300060, China; ^2^Key Laboratory of Breast Cancer Prevention and Therapy, Tianjin Medical University, Ministry of Education, Tianjin 300060, China; ^3^Key Laboratory of Cancer Prevention and Therapy, Tianjin 300060, China; ^4^Tianjin's Clinical Research Center for Cancer, Tianjin 300060, China; ^5^Department of General Surgery, Tianjin Medical University General Hospital, Tianjin 300052, China

## Abstract

**Background:**

Thyroid cancer (TC) tends to be a common malignancy worldwide and results in various outcomes due to its different subtypes. The tumor microenvironment (TME) was demonstrated to play crucial roles in various malignancies, including thyroid cancer. This study combined the ESTIMATE and CIBERSORT algorithms, identified four TME-related genes, and evaluated their correlation with clinical characteristics. These findings revealed the malignant performance of TME in TC, and the TME-related DEGs might serve as prognostic biomarkers, which can be utilized for the prediction of immunotherapy effects in patients with TC.

**Methods:**

The clinical and gene expression profiles of TC patients were collected from the TCGA dataset. The ESTIMATE algorithm was utilized to estimate stromal and immune scores and predict the level of stromal and immune cell infiltration. The differential expressed genes related to TME were filtered by the “limma” package in R, and the PPI network was constructed by a string website. KEGG pathway and GO analyses were performed to investigate the biological progression and molecular functions of TME-related DEGs. Then, univariate Cox regression analysis was employed to screen four genes correlated with clinical characteristics. GSEA was conducted to assess their roles in the TME of TC. To further investigate the association between TME-related genes and tumor-infiltrating immune cells (TIICs), the CIBERSORT algorithm was performed. Finally, the malignancy behaviors of the two genes were verified by RT-qPCR, IHC, MTT, colony formation, and transwell assays.

**Results:**

Four TME-related DEGs, LRRN4CL, HS3ST3A1, PCOLCE2, and CAPN8, were identified and were significantly predictive of poor overall survival. KEGG and GO pathway analysis established that the TME-related DEGs were involved in immune responses and pathways in cancer. Furthermore, the malignancy behaviors of HS3ST3A1 and CAPN8 were verified by cellular functional experiments. These results revealed that the TME-related genes HS3ST3A1 and CAPN8 were able to serve as predictors of prognosis in patients with TC.

**Conclusion:**

HS3ST3A1 and CAPN8 may serve as valuable prognostic biomarkers and TME indicators, which can be utilized for the prediction of immunotherapy effects and provide novel treatment strategies for patients with TC.

## 1. Introduction

Thyroid cancer (TC) occurs commonly worldwide and represents 3% of the global incidence of all cancers, which shows a continuously increasing in the past three decades with 586000 new patients in 2020 [1, 2]. In females, it develops three or four times more frequently but is less destructive than in males [3, 4]. The classification of TC subtypes is according to their differentiated degree, which was commonly classified into 4 histological types. Follicular thyroid carcinoma (FTC) and papillary thyroid carcinoma (PTC) are well-differentiated thyroid cancers (WDTC) with better prognosis, while there are still poorly-differentiated thyroid cancer (PDTC) and anaplastic thyroid carcinoma (ATC) [5]. PTC is the most common subtype that occupies nearly 80% of DTC [6]. The initial management of DTC includes surgical resection, radioactive iodine ablation (RAI), and thyroid-stimulating hormone (TSH) suppression, after which, patients usually owe good outcomes. However, 10–15% of patients with thyroid cancer have recurrent disease, 5% develop distant metastasis (lungs and bones), and cancer-specific death occurs in some cases [7]. Thus, it is essential to explore emerging biomarkers that contribute to prognosis prediction in thyroid cancer.

In previous studies, it has been demonstrated that the tumor microenvironment (TME) is intimately correlated to the development and prognosis of multicancer types [8–10]. Therefore, increased attention has been paid to the role of initiation and progression that TME plays in cancer. Stomal and immune cells stand for two primary nontumor members in TME, while there are still other complex components, including extracellular matrix (ECM) and inflammatory mediators [11]. Emerging evidence has indicated that tumor-infiltrating immune cells (TIICs), for instance, regulatory T cells (Tregs), CD8+ T cells, CD4+ T cells, and tumor-associated macrophages (TAMs), are involved in various malignancies [12, 13]. Despite the previous study identifying several critical genes related to TME in thyroid cancer, further investigations are still necessary [14].

Estimation of Stromal and Immune cells in Malignant Tumor tissues using Expression data (ESTIMATE) is a standard algorithm for calculating scores of immune and stromal. Here, we utilized the ESTIMATE algorithm to calculate immune and stromal scores for patients with TC based on the gene expression profiles from The Cancer Genome Atlas (TCGA). Then, the core genes involved in clinical outcomes were identified via univariate Cox regression analysis. In this way, we expect to find out emerging genes, which can perform as biomarkers of prognostic prediction for patients with TC.

## 2. Materials and Methods

### 2.1. Data Collection and Mining

The gene expression and clinical profiles of TC patients were collected from the TCGA dataset (https://portal.gdc.cancer.gov/). The filtration conditions were set as “thyroid gland” and “TCGA-THCA”. Finally, 568 patients with TC (58 normal specimens and 510 cancer specimens) were selected. The composition of the TME in these specimens was evaluated by the ESTIMATE algorithm, and the results were shown as stromal score, immune score, and estimate score. “limma” package in R was performed to screen differential expressed genes (DEGs) with criteria as follow: (1) |log 2FC| >  1; (2) FDR <0.05. After that, the “heatmap” package in R was employed to make TME-related DEGs visualization as heatmaps, and the “VennDiagram” package in R was employed to select similar genes in immune and stromal cells.

### 2.2. Functional Analysis of DEGs

Kyoto Encyclopedia of Genes and Genomes (KEGG) and Gene Ontology (GO) analysis had been employed in TME-related genes. The “clusterProfiler,” “enrichplot,” and “ggplot2” packages were utilized for the visualization of KEGG and GO analysis results, which showed the biological process, molecular function, and pathways of TME-related DEGs. Data with *P*  <  0.05 and *q* < 0.05 were considered with statistical significance.

### 2.3. PPI Network and Cox Analysis of DEGs

The STRING database (https://cn.string-db.org/) was employed to construct an interaction network of TME-related DEGs. Interactions with integrated scores higher than 0.95 were selected and visualized by Cytoscape (version 3.8.2). The “survival” package in R was utilized to perform univariate Cox regression to determine the association of DEGs with the prognosis of TC. Ultimately, four core genes were screened to perform the following analysis.

### 2.4. Analysis of Tumor Immunoreaction

To investigate the capable pathway that TME-related DEGs act on in the TME of TC, the Gene Set Enrichment Analysis (GSEA) was conducted using GSEA version 4.1.0 (Broad Institute, Cambridge, MA, United States). The statistical significance was set as NOM *P* value <0.05 and FDR <0.25. The correlation between TME-related DEGs and TIICs was assessed by the CIBERSORT algorithm, which is a deconvolution algorithm based on RNA-seq data to estimate the proportion of 22 immune cells in each specimen.

### 2.5. Correlation between TME-Related DEGs and Clinicopathological Characteristics

The overall survival (OS) was compared between the TC samples with high/low expression of TME-related DEGs through Kaplan-Meier survival analysis. Furthermore, the correlation analysis between DEGs and clinicopathological characteristics was performed, and results with *P*  <  0.05 and *q* < 0.05 were considered with statistical significance.

### 2.6. RNA Extraction and RT-qPCR

Extraction of total RNA was performed by RNAprep pure Tissue Kit (Tiangen Biotech, Beijing). The RNA reverse transcription was performed by *TansScript*® All-in-One First-Strand cDNA Synthesis SuperMix for qPCR (Transgen, China), and RT-qPCR was conducted using *TransStart*® Green qPCR SuperMix (Transgen, China) according to standard protocols. The primer sequences of RT-qPCR were exhibited in the supplementary file: Table S1.

### 2.7. Immunohistochemistry (IHC) Staining

The paraffin-embedded sections of thyroid cancer tissue and normal tissue were deparaffinized, rehydrated, and antigen-retrieved with sodium citrate buffer (10 mM, pH 6.0), then incubated with antibodies of HS3ST3A1 or CAPN8 (supplementary file: Table S2) at the dilution of 1 : 100 overnight. Then, the sections were incubated with corresponding secondary antibodies at 1 : 500 dilutions (Selleck, USA), and stained with 3,3′-diaminobenzidine (DAB) (Sigma, USA). After being mounted, the slides were observed and captured under a microscope (Olympus, Japan). The percentage of stained cells and the staining intensity were calculated using criteria as follows: (a) percentage of stained cells: 4 (>75%), 3 (51%–75%), 2 (26%–50%), 1 (1%–25%), and 0 (0%); (b) staining intensity: 3 (strong staining), 2 (moderate staining), 1 (weak staining) and 0 (negative staining). The scores were shown as scatter plots.

### 2.8. Cell Culture

The papillary thyroid cancer cells BPCAP and TPC-1 were purchased from the Cell Bank of the Chinese Academy of Sciences (Shanghai, China) and cultured with DMEM (Solarbio, China) and RPMI 1640 (Solarbio, China), respectively, in a 37°C incubator with 5% CO_2_. All mediums were added 10% fetal bovine serum (FBS) (AusGeneX, Australia) and 1% penicillin/streptomycin (Solarbio, China).

### 2.9. Transfection of Short Hairpin RNAs (shRNAs)

The shRNAs of HS3ST3A1 and CAPN8, as well as their control groups, were obtained from RiboBio (Guangzhou, China), which sequences were exhibited in the supplementary file: Table S3. The transient transfection was conducted according to the standard protocol of FuGENE® HD Transfection Reagent (Promega, USA).

### 2.10. MTT and Colony Formation Assay

The MTT assay was performed by seeding 2 ∗ 10^3^ cells in 96-well plates after transfection for 48 h. The 3-(4,5-dimethylthiazol-2-yi)-2,5-diphenyltetrazolium bromide (MTT) was used to assess cell proliferation, and the absorbance was detected by Tecan A-5082 sunrise (Austria) at the same time points from day 1 to day 5. The colony information assay was performed by seeding 5 ∗ 10^2^ cells in 6-well plates after transfection for 48 h. After being cultured for 2-3 weeks, the colonies were fixed and stained.

### 2.11. Transwell Assay

The transwell assay was performed by seeding 5 × 10^4^ cells in the upper chambers with 10% FBS medium, while the lower chambers were added with 20% FBS medium. After incubating for 10–14 h., the cells in the upper chamber were fixed and stained by a three-step set (Thermo Scientific, USA). Images were taken by a light microscope (Olympus, Japan) at 100^*∗*^ magnification, the migrated cells were counted and calculated.

### 2.12. Western Blot

RIPA buffer (Solarbio, China) with PMSF (Thermo Scientific, USA) was utilized to extract cellular proteins, and the concentration was detected using a BCA kit (Thermo Scientific, USA). The 10% SDS-PAGE gels were used for protein separation and then the separated proteins were transferred to PVDF membranes. The 5% skimmed milk block was applied for one hour and then incubated at 4°C overnight with diluted primary antibodies. After three times washing with TBST, the membranes were incubated with secondary antibodies for one hour at room temperature. Finally, the blots were detected by ECL (Millipore, USA). The antibodies information was listed in the supplementary file: Table S2.

### 2.13. Statistical Analysis

Mean and standard deviation (SD) were utilized for presenting data. The significance of differences between the experimental and control groups was determined by Student's *t*-test. Results with *P*  <  0.05 were considered with statistical significance. The calculations of all data were conducted using IBM SPSS software (version 22.0, USA).

## 3. Results

### 3.1. Identification and Functional Analysis of DEGs

**Figure 1:**
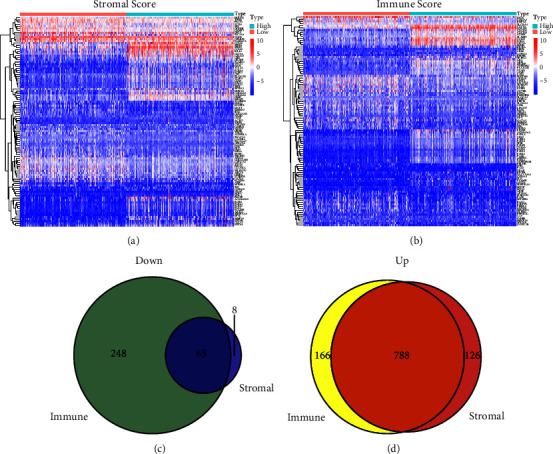
The heatmaps and Venn plots of differential expressed genes (DEGs). (a) The heatmap of stromal-related genes with high-and low-expression groups based on the media score. (b) The heatmap of immune-related genes with high- and low-expression groups based on the media score. (c) The Venn plot of commonly downregulated DEGs in the stromal and immune components. (d) The Venn plot of commonly upregulated DEGs in the stromal and immune components.

**Figure 2:**
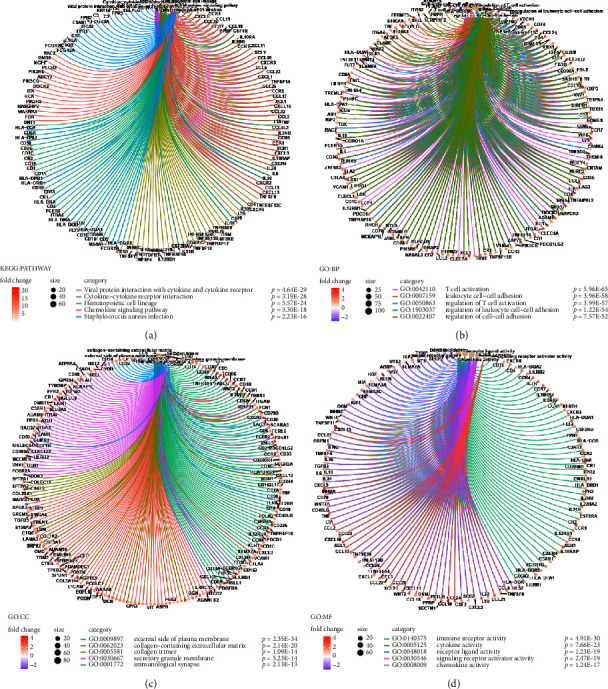
Enrichment analysis of DEGs. (a) Kyoto Encyclopedia of Genes and Genomes (KEGG). Gene ontology (GO) analysis, including (b) biological processes (BP), (c) cellular components (CC), and (d) molecular functions (MF).

To identify differentially expressed genes related to TME, the specimens were divided into high- and low-score groups according to stromal score, immune score, and ESTIMATE score. Finally, 987 DEGs were identified according to stromal score, containing 914 upregulated genes and 73 down-regulated genes (Figures 1(a), 1(c) and 1(d)). Correspondingly, 1267 DEGs were obtained according to the immune score, including 954 upregulated genes and 313 downregulated genes (Figures 1(b)–1(d)). The Venn plot was used to identify 788 upregulated genes and 65 downregulated genes in both the immune and stromal components (Figures 1(c) and 1(d)). Then, these 853 DEGs were considered TME-related DEGs. To further investigate the biological functions and capable pathways associated with these TME-related DEGs, the analyses of KEGG and GO were performed. The results of KEGG analysis shows that DEGs were involved in some immune-related activities, for instance, cytokine-cytokine receptor interaction and chemokine signaling pathway (Figure 2(a)). While the results of GO analysis, which contains molecular functions (MF), biological processes (BP), and cellular components (CC), indicated that the TME-related DEGs were primarily enriched in immune-related functions, including GO:0042110 (T-cell activation), GO:0001772 (immunological synapse), and GO:0140375 (immune receptor activity) (Figures 2(b)–2(d)).

### 3.2. PPI Network and Univariate Cox Regression Analysis

**Figure 3:**
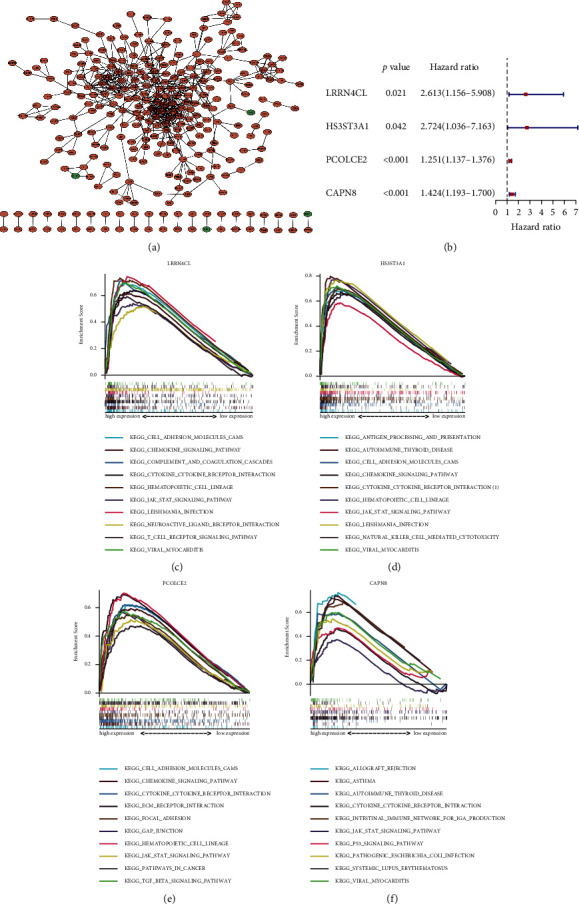
Protein–protein interaction (PPI) network, univariate Cox regression analysis, and GSEA enrichment analysis. (a) Construction of the PPI network of 853 differentially expressed genes (DEGs), upregulated genes were shown in red, while downregulated genes were in the green. (b) Results of univariate Cox regression analysis with selected DEGs identified four genes and displayed them in the forest plot. The gene set enrichment analysis (GSEA) of (c) LRRN4CL, (d) HS3ST3A1, (e) PCOLCE2, and (f) CAPN8.

After obtaining TME-related DEGs through the above analyses, the PPI network had been constructed using the String dataset to investigate the interactions among these genes and the result was visualized using Cytoscape (Figure 3(a)). Then, the univariate Cox regression analysis was performed to explore the correlation between DEGs and the prognosis of patients with TC. Four genes were identified as risk factors of TC prognosis (Figure 3(b)), which were set as the main characters of our following analysis. To further investigate their functions, GSEA was conducted among these four DEGs, which indicated that they were largely engaged in immune-related events, such as the JAK-STAT signaling pathway, T cell receptor pathway, and autoimmune thyroid diseases. Besides, it is worth mentioning that specific enrichment results also showed the significant role of these genes in cancer, including the P53 signaling pathway, pathways in cancer, and TGF-*β* signaling pathway (Figures 3(c)–3(f)).

### 3.3. The Role of 4 Core DEGs in TME of TC

**Figure 4:**
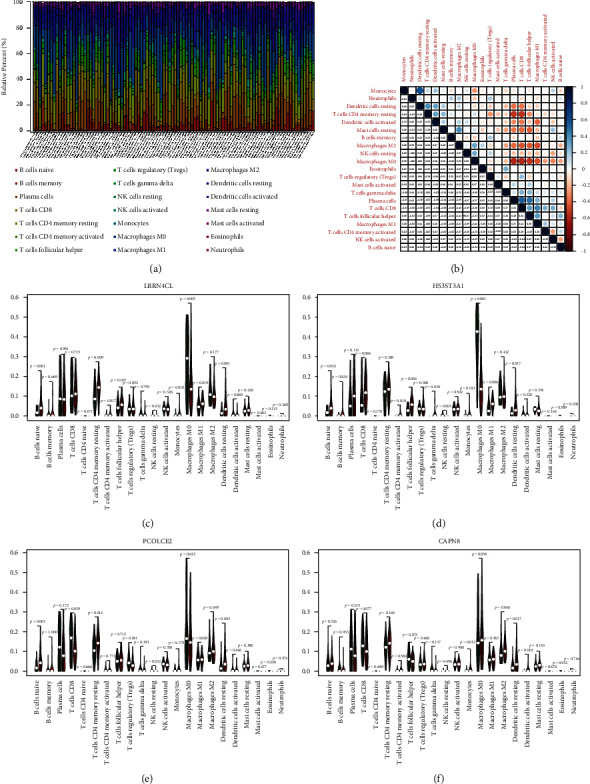
Tumor-infiltrating immune cells (TIICs) in TC samples and their correlation analysis. (a) Barplot showing the proportion of 21 different types of TIICs in TC samples. (b) The heatmap shows the correlation between 21 different types of TIICs. The violin plot shows the differences in the proportions of 21 different types of immune cells in TC samples with high- or low-expression of core genes. (c) LRRN4CL, (d) HS3ST3A1, (e) PCOLCE2 and (f) CAPN8.

**Figure 5:**
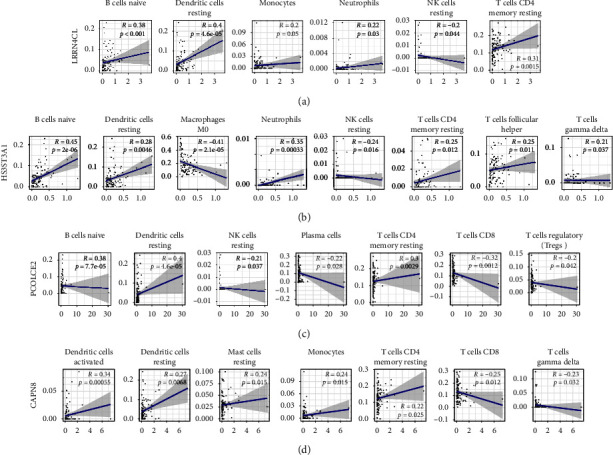
The scatter plot shows the correlation of the proportions of TIICs. (a) LRRN4CL, (b) HS3ST3A1, (c) PCOLCE2, and (d) CAPN8.

Then, the infiltrating data of 22 different types of immune cells in tumor tissues was identified using the CIBERSORT algorithm (Figures 4(a) and 4(b)). The different types of TIICs were discovered to be closely involved in varying core gene expression in the TME of TC cells, and detailed results were established as violin plots (Figure 4(c)). The interrelation between the proportions of TIICs and core DEGs expression was shown as the scatter plots, respectively, including LRRN4CL (Figure 5(a)), HS3ST3A1 (Figure 5(b)), PCOLCE2 (Figure 5(c)), and CAPN8 (Figure 5(d)), which showed these 4 core genes were positively correlated with dendritic cells (DCs), monocytes, (MC) and neutrophils, while negatively associated with NK cells, plasma cells, CD8+ T cells, and M0 macrophages. Therefore, these results indicated that LRRN4CL, HS3ST3A1, PCOLCE2, and CAPN8 play a crucial role in the immune activities of TC cells.

### 3.4. The Correlation between 4 Core DEGs and Clinical Signatures

**Figure 6:**
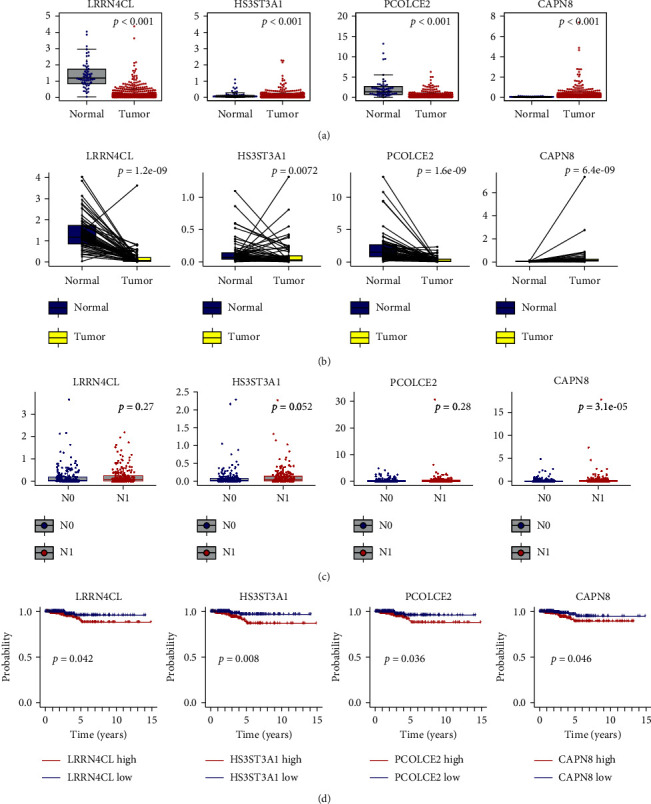
The expression levels of 4 core DEGs in clinical specimens: (a) unpaired and (b) paired. (c) The correlation between four core DEGs and N stage. (d) The correlation between four core DEGs and overall survival (OS) of TC.

In the previous study, the core DEGs were identified as LRRN4CL, HS3ST3A1, PCOLCE2, and CAPN8. The TC specimens were divided into high- and low-groups according to the expression of core DEGs, then we investigated the role of these core genes in the clinical outcome of TC patients, which showed that all these 4 DEGs were related to poor OS in patients with TC (*P*=0.042,*P*=0.008, *P*=0.036, and *P*=0.046, respectively) (Figure 6(d)). Additionally, the expressions of these DEGs were relatively associated with the N stage, especially for CAPN8 (*P*=3.1*e* − 05) (Figure 6(c)). The Wilcoxon rank-sum test indicated the expression levels of 4 DEGs among normal and tumor tissue in paired or unpaired samples, which indicated HS3ST3A1 and CAPN8 expressed significantly higher in tumor tissues, while the other two genes, LRRN4CL and PCOLCE2, were not (Figures 6(a) and 6(b)). Therefore, HS3ST3A1 and CAPN8 were selected for further investigation to verify their malignancy behavior in experiments.

### 3.5. HS3ST3A1 and CAPN8 Were Associated with Higher Tumor Stage

**Figure 7:**
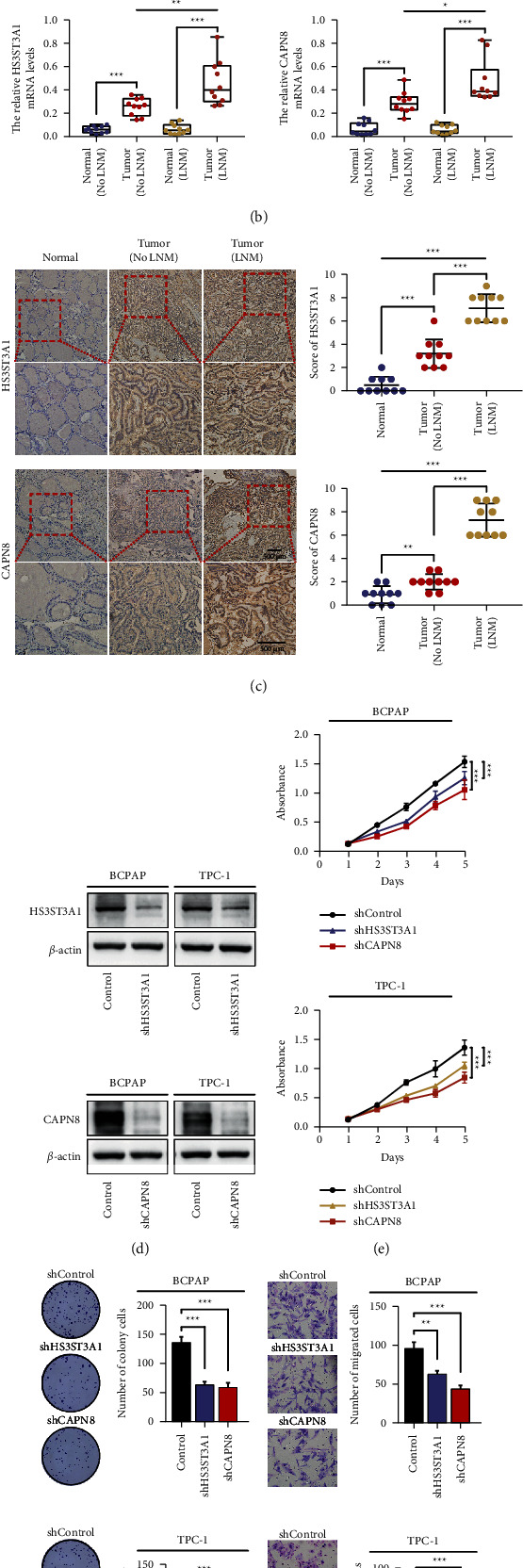
The expression of HS3ST3A1 and CAPN8 in clinical samples and cellular functional experiments. (a) GEPIA analysis show HS3ST3A1 and CAPN8 differed among stages. (b) RT-qPCR of HS3ST3A1 and CAPN8 in 20 paired TC tissues, including 10 paired samples with LNM and 10 paired samples with no LNM. (c) IHC staining of HS3ST3A1 and CAPN8 in 30 TC specimens, including 10 adjacent normal samples, 10 tumor samples with no LNM, and 10 tumor samples with LNM. (d) Western blot of downregulation of HS3ST3A1 and CAPN8 in papillary TC cells BPCAP and TPC-1. The effect of HS3ST3A1 and CAPN8 downregulation on proliferation and invasion is determined by (e) MTT, (f) colony formation, and (g) Transwell. ^*∗*^*p*  <  0.05, ^*∗∗∗*^*p*  <  0.01.

The GEPIA (https://gepia.cancer-pku.cn) was employed for further investigation of the correlation between HS3ST3A1 or CAPN8 with clinical stages, which results showed that both HS3ST3A1 and CAPN8 significantly differed among stages (Figure 7(a)). In our previous study, the expression of DEGs seemed to be inextricably linked to the N stage. Therefore, we collected 10 paired samples of thyroid cancer with lymph node metastasis (LNM) and nonlymph node metastasis (No LNM), respectively. RT-qPCR results indicated that the mRNA levels of HS3ST3A1 and CAPN8 were remarkably higher in tumor tissues compared with normal tissues. Furthermore, their mRNA expression levels in tumors with LNM were notably higher compared with those without LNM (Figure 7(b)). Then, we collected 30 cases of TC patient tissues, including normal, tumors with LNM and tumors with no LNM. The IHC results indicated that HS3ST3A1 and CAPN8 expression levels were both higher in tumors with LNM than in tumors with no LNM or normal tissues (Figure 7(c)). These findings indicated that HS3ST3A1 and CAPN8 were intimately correlated to higher clinical stages and lymph node metastasis.

### 3.6. Downregulation of HS3ST3A1 or CAPN8 Inhibit Malignancy Behaviors of Thyroid Cancer Cells

To verify the cellular function of HS3ST3A1 and CAPN8, we transfected shHS3ST3A1 and shCAPN8 into papillary thyroid cancer cells BCPAP and TPC-1, which downregulation was detected by Western blot (Figure 7(d)). Furthermore, cells with shHS3ST3A1 and shCAPN8 showed less proliferation rates (Figure 7(e)) and generated fewer colonies (Figure 7(f)) than the control groups. As for the transwell assay, cells with shHS3ST3A1 and shCAPN8 also behaved less aggressively (Figure 7(g)). These results demonstrated that the downregulation of HS3ST3A1 and CAPN8 was able to inhibit the proliferation and invasion of thyroid cancer cells in vitro.

## 4. Discussion

It has been reported in a huge number of studies that TME plays critical roles in multiple cancer types and promotes the progression of molecular classification systems and treatment strategies [15]. Our study identified LRRN4CL, HS3ST3A1, PCOLCE2, and CAPN8 as TME-related genes associated with prognosis in TC samples collected from the TCGA database, and verified two of them, HS3ST3A1 and CAPN8, through a clinical specimen and cellular functional experiments.

LRRN4CL lacks in-depth research, while only one study reported that the upregulation of the cell surface protein LRRN4CL promoted metastases of pulmonary in mice [16]. HS3ST3A1 encodes the enzyme 3-O-sulfotransferase, which catalyzes the biosynthesis of 3-O-sulfated heparan sulfate, a specific subtype of heparan sulfate (HS). It has been reported that HS3ST3A1 is involved in respiratory papillomatosis [17] and human immunodeficiency virus (HIV) infection [18]. Besides, HS3ST3A1 was found to be upregulated in lung cancer tissue compared with normal lung tissue and associated with the progression of lung cancer [19]. PCOLCE2 is reported to encode a functional collagen-binding protein procollagen C-proteinase enhancer (PCPE2) [20], and evidence has proved that PCOLCE2 was able to perform as a biomarker for prognostic prediction in colorectal cancer patients [21]. CAPN8 belongs to the calpain family and exhibits restricted expression patterns [22] and has been proposed to be involved in vesicle trafficking [23]. However, there are no investigations on the role of these four genes in thyroid cancer. Synthesizing previous studies, there are hardly any reports on their functions in the tumor microenvironment, but their roles as prognosis indicators are certainly clarified in several types of cancer. It is our study that collects LRRN4CL, HS3ST3A1, PCOLCE2, and CAPN8 all together for the first time and sets them as biomarkers related to TME in thyroid cancer, which might become potential candidate targets for TC immunotherapy.

It is commonly recognized that inflammation and autoimmunity are risk factors for TC [24]. Evidence also indicated that TC patients might benefit from targeting cancer-related inflammation, which provided new strategies for diagnosis and treatment [25]. Studies of immune infiltration in TC have made many major advances, particularly in the study of primary immune cells, such as NK cells, TAMs, MCs, DCs, CD8+ T cells, B cells, neutrophils, and Tregs. [24, 26, 27]. In our study, the proportion of TIICs was estimated using the CIBERSORT algorithm, which indicated that TME-related DEGs had a notable association with specific immune cells, including monocytes, neutrophils, T cells, and so on. Previous studies revealed that TAMs, MCs, DCs, Tregs, and neutrophils were positively related to TC progression [28–32]. While NK cells, iDCs, CD8+ T cells, and B cells are negatively associated with TC progression [33–38]. However, some studies clarified that immune cells are related to various outcomes in cancer [39]. It is sure that further studies will be conducted to explore the role of different immune cells plays in cancer for better prognostic evaluation.

As for TME-related genes in thyroid cancer, a previous study has identified 30 hub genes by constructing the PPI network and set CXCL10 as the top hub gene [14]. Our study has different logical methods and conducted further data mining. First, we obtained more gene expression and clinical profiles so that our study might have more credibility. CXCL10 was a specific differential expressed TME-related gene in the PPI network with the most nodes, but it tended to lose its significance when performing univariate Cox regression analysis. Furthermore, we analyzed the immune infiltrating profiles of TC and concluded the correlation between immune cells and DEGs using the CIBERSORT algorithm. These make sense for better identifying TME-related biomarkers and prognostic indicators in patients with TC.

HS3ST3A1 and CAPN8 were considered to serve as prognostic predictors in our study. Clinical specimens were collected and subjected to RT-qPCR and IHC staining, which showed higher HS3ST3A1 and CAPN8 expression levels in tumor tissues, especially in tumors with LNM. In vitro, the downregulation of HS3ST3A1 and CAPN8 presented the inhibition of proliferation and invasion in papillary thyroid cancer cells. These findings indicated that TME-related genes HS3ST3A1 and CAPN8 function in the immune process and contribute to tumor development.

However, it is necessary to acknowledge the limitations of this study. The biases were unavoidable because the data were primarily obtained from the TCGA database. Besides, we chose two of the genes for experimental verification; the other two genes, LRRN4CL and PCOLCE2 were excluded due to their adverse expression both in paired and unpaired tissues. As for clinical specimens, our data lacks survival information due to the good outcome of TC patients. Furthermore, our experiments mainly focused on papillary thyroid cancer cells, which is the most common subtype of TC, but the verification in other thyroid cancer subtypes is lacking. Animal experiments and the depth of molecular mechanisms still need further investigation.

Overall, we used the ESTIMATE algorithm to identify DEGs related to the TME in TC specimens obtained from the TCGA dataset. LRRN4CL, HS3ST3A1, PCOLCE2, and CAPN8 were observed as potential prognostic indicators for patients with TC. Furthermore, HS3ST3A1 and CAPN8 were highly expressed in thyroid tumor tissue, especially in tumors with LNM, and their downregulation can inhibit the proliferation and invasion of thyroid cancer cells. However, the underlying molecular mechanisms of tumor micro-metastasis and the potential clinical value for early diagnosis still require further experimental study.

## 5. Conclusion

In summary, we identified several TME-related DEGs in TC, among them, LRRN4CL, HS3ST3A1, PCOLCE2, and CAPN8 were remarkably involved in the regulation of the immune activities in the TME and poor clinical outcomes. Additionally, the malignancy behaviors of HS3ST3A1 and CAPN8 in the tumor process were verified through tissue and cellular experiments. Our findings indicated that HS3ST3A1 and CAPN8 served as prognostic biomarkers of TC and might bring new insights into the development of effective therapeutic strategies for patients with TC.

## Data Availability

All data generated or analyzed during this study are included in this article and its supplementary files.

## Supplementary Materials

Supplementary Materials
*Table S1.* The sequences of primers used for RT-qPCR. *Table S2.* The antibodies used for IHC and Western blot. *Table S3.* Target sequences of shRNA.
