# Supplementary material for: HS3ST3A1 and CAPN8 Serve as Immune-Related Biomarkers for Predicting the Prognosis in Thyroid Cancer
Source: J Oncol. 2022 Dec 22;2022:6724295. doi: 10.1155/2022/6724295 (PMC9800087; doi:10.1155/2022/6724295)
Supplement: Supplementary Materials — Table S1. The sequences of primers used for RT-qPCR. Table S2. The antibodies used for IHC and Western blot. Table S3. Target sequences of shRNA. [file 6724295.f1.docx]

**Table S1** Primers used for RT-qPCR

| **Gene name** | **Forward Primer (5’ to 3’)** | **Reverse Primer (5’ to 3’)** |
| --- | --- | --- |
| HS3ST3A1 | CCCTGCTCACGTCCCTTTAC | CAGGAGGCGCTTTCTCTGTG |
| CAPN8 | TGGCTCCAACCAAAACGCTT | CCTGGTCCAAGATCCTTGTAGC |

**Table S2** Antibodies used for IHC and western blot

| **Antibodies** | **Source** | **Company** |
| --- | --- | --- |
| HS3ST3A1 | Rabbit | Bioss |
| CAPN8 | Rabbit | ABCEPTA |
| β-actin | Mouse | Cell Signaling Technology |

**Table S3** Target sequence of shRNA

| **Target gene (human)** | **Target sequence (5’ to 3’)** |
| --- | --- |
| shHS3ST3A1 | GCAGCATCTTCCGGAAGTT |
| shCAPN8 | CCCTGACCCTGAATGAAGA |
